# A Nodulation-Proficient Nonrhizobial Inhabitant of* Pueraria phaseoloides*

**DOI:** 10.1155/2019/9782684

**Published:** 2019-04-01

**Authors:** W. M. M. Wedage, A. H. M. N. R. Aberathne, I. N. Harischandra, D. Gunawardana

**Affiliations:** ^1^Department of Botany, Faculty of Applied Sciences, University of Sri Jayewardenepura, Sri Lanka; ^2^Centre for Biotechnology, Department of Zoology, Faculty of Applied Sciences, University of Sri Jayewardenepura, Sri Lanka; ^3^Genetics and Molecular Biology Unit, Faculty of Applied Sciences, University of Sri Jayewardenepura, Sri Lanka

## Abstract

*Pueraria phaseoloides* is a legume cover crop, found chiefly in the wet zone of Sri Lanka. Nitrogen fixation is performed by nodular inhabitants of this cover crop, comparable to the nodule-dwelling bacteria of most other legume plants. We isolated a bacterium (Sub1) from* Pueraria phaseoloides*, of coccobacillus cell shape, that showed nodulation, when assessed by hydroponics, showing nodules as early as 3 weeks after reinfection. When a* nifH* fragment from the genome of this bacterium was amplified using a pair of* nifH* primers, it yielded an amplicon of 360 bp that, when sequenced, helped us identify the bacterium, as belonging to a species of* Pseudacidovorax intermedius,* at 99% sequence identity. When we constructed a phylogenetic tree with neighboring sequences, we encountered* nifH *sequences of* Pseudacidovorax,* forming a monophyletic cluster, which too contained a single* Azospirillum *species. The genus* Pseudacidovorax* is a bacterium that, so far, has not been associated with legume nodules. Sub1 secreted a pair of enzymes to the extracellular medium to degrade cellulose and milk proteins. The Sub1 bacterium showed biofilm formation and secreted into the extracellular medium, indole acetic acid. Sub1 also showed a “bulls eye” swarming pattern for the chemoattractant proline, while showing no significant chemotaxis movement, for naringenin, quercetin, and glutamate. Sub1 too possesses the basic genetic foundation (*nifH* and* nifD*) to produce a molybdenum-dependent nitrogenase enzyme. We finally show that this rare nonrhizobial bacterium is able to impact, positively, nodulation and shoot length of* Pueraria *plants, demonstrating that this beta-proteobacterium can abet the biological vigor of this legume cover crop.

## 1. Introduction

Nitrogen is a macronutrient for plant growth. It plays mandatory roles inside plant cells for the synthesis of enzymes, proteins, chlorophyll, DNA, and RNA [1]. Rubber is one of the major industrial crops in Sri Lanka, especially in the lowlands, where nutrient enrichment through cultivation of cover crops, ensures optimum latex yields. In the contemporary, the use of leguminous cover crops such as* Pueraria phaseoloides* is a widely seen practice, in rubber plantations. Pueraria is called “Pohora Wal” (Fertilizer Vines) in Sri Lanka and is a common occupant in rubber plantations, which makes this an invaluable cover crop of economic importance.

Legumes emerged 70-130 million years ago early in the Cretaceous period [2]. Legumes possess root nodules which harbor bacteria of the genus* Rhizobium*, which fosters an advantage from the nitrogen enrichment, converting a large reservoir of nitrogen gas, into amino acids [3] While Rhizobia is host specific, we do need to know the status of nonrhizobial inhabitants that live inside legume nodules, whether they are, too, host specific, and their impact on plant growth [4].

*Pueraria phaseoloides, *which is also known by the names, Puero (Australia), tropical kudzu (most of the tropics), centro grande, and feuille, is a cover crop that grows well in wet land soils, and supplies the voracious needs of the root system of rubber plants. One of the predominant features of* Pueraria phaseoloides* is the presence of a wide range of nitrogen-fixing bacteria inside the root nodules [5]. This supposed ‘promiscuous' nature of nodular life makes* Pueraria phaseoloides *the ideal legume to investigate its compliment of nonrhizobial nitrogen fixers.

Several nodulation-competent nonrhizobial species, belonging to *α* and *β* subgroups of Proteobacteria such as* Methylobacterium, Blastobacter, Devosia, Phyllobacterium, Ochrobactrum, Agrobacterium, Cupriavidus, Herbaspirillum, *and* Burkholderia* and some *δ*-Proteobacteria have been identified this far [2]. Although universally nitrogen fixation and nodulation genes have low divergence tendencies, particular attention has been paid on some nodular nonrhizobial nitrogen-fixers due to significant sequence divergences in the nitrogen fixing (*nifH*) and nodulation (*nodD* or* nodA*) gene sequences [2].

We describe here, a rare nonrhizobial nitrogen fixer, residing inside nodules of* Pueraria phaseoloides *using a combination of molecular biology, microbiology, and plant growth experiments.

## 2. Methods

### 2.1. Isolation of Nodule-Inhabiting Bacteria

Surface sterilized [8] root nodules were crushed by using sterile mortar and pestle while mixing with sterilized distilled water and Yeast Mannitol Agar containing petri plates were streaked with the macerate and incubated at room temperature for 1-2 days.

### 2.2. Optical Microscopy

To study cell morphology, single colonies from agar plates were used to prepare slides and the prepared slides were stained with simple staining and Gram staining [9] techniques and observed under 4×, 10×, 40×, 100× magnifications (Light Microscopy).

### 2.3. Assays for Cellulose, Pectin, and Protein Utilization

For cellulose, pectin, and protein utilization, media plates were made with carboxy methyl cellulose, pectin, and skim milk and the hydrolysis zones around the bacterial colonies were observed/measured to identify the respective hydrolysis-promoting bacteria [10–12].

### 2.4. Biofilm Assay

Cultures in YMB medium were incubated overnight at 150 rpm shaker. 12 boiling tubes were autoclaved by setting cotton plugs and aluminum foil. Overnight cultures were diluted 1:50 (100 *μ*l of culture with 5ml of medium) and incubated 3-5 days. Cultures were removed carefully using a pipette and the boiling tube was washed 3-5 times with two times autoclaved distilled water. Then, 2ml of 0.5% crystal violet was added to each tube. After incubation of 20 minutes, crystal violet was removed and tubes were washed with two times autoclaved distilled water, 5 times. Tubes were turned upside down and they were dried overnight. Then, 2ml of 30% acetic acid in water was added to each tube and the tubes were incubated at room temperature for 10-15 minutes. Finally, absorbance was measured at 550 nm using 30% acetic acid in water as a blank [13].

### 2.5. Chemotaxis (Swarming) Assays

Petri pates were prepared by adding MA medium and ammonium chloride was added as the nitrogen source. 10mM L-glutamic acid solution was prepared separately and autoclaved. A filter paper disc, which was dipped in bacteria broth, was placed in one side of the plate and another disc, which was dipped in 10mM glutamic acid, was placed in the opposite side of the plate. The petri plates were incubated at room temperature and movements were observed. The same was done for 10mM naringenin (positive flavonoid) and 10mM quercetin (negative flavonoid) [16].

Furthermore, culture media were prepared with 0.3% of bacto agar, with 10^−4^M proline as the chemoattractant and mannitol as the energy source and the chemotaxis-based migration (swarming patterns) of bacteria were recorded after 4 days of incubation at room temperature [17].

### 2.6. IAA Assays

Bacterial isolates were cultivated in YEM broth supplemented with 5 mM L-Tryptophan at 30°C for 05 days. A control was also maintained without inoculating bacteria. Then 1ml aliquots of each media were centrifuged at 10,000 rpm for 12 minutes after 24, 48, and 72 hours of growth (until 5 days). Then 1ml of sample was treated with 2ml of Salkowski's reagent in a test tube (to prepare 100ml of Salkowski's reagent, 2 ml of 0.5 M FeCl_3_, 49 ml of water, and 49 ml of 70% perchloric acid were mixed together). Resulting solution was analyzed using spectrophotometer at 530 nm. IAA concentration of each sample was calculated using standard curve of commercial IAA.

Standards were prepared in YEM at 1, 5, 10, 20, 50, and 100 ppm and to prepare the 1000 ppm stock solution and 10 mg of IAA was dissolved in 10ml of acetone and stirred well. A series of vials with the dilution series were labeled. Then, 100 ppm solution was prepared by adding 1ml of 1000 ppm stock to the 9ml of YEM medium and mixed by inversion. The dilutions were prepared accordingly.

Then, 2 ml of Salkowski's reagent was added to 1 ml of each standard in a test tube including a control without IAA. They were allowed to develop the color at room temperature and absorbance was read at 530 nm. After entering data on an Excel sheet, a graph was drawn. Afterwards, the concentration of IAA produced by each bacterium was calculated [18].

### 2.7. PCR Amplification of* nifH* and* nifD* Gene Fragments

Polymerase Chain Reactions were run with the PolF and PolR primers and nifD_F and nifD_R as described in Poly et al. (2001). The primers used were as shown in Table 1.

### 2.8. Sequencing of* nifH* Gene Fragments and Phylogenetic Reconstruction

PCR amplified products for* nifH* gene fragments were purified and sequenced bidirectionally by Sanger sequencing method. Sequences were assembled using DNA baser (V 4.20) software. Open Reading Frames (ORFs) were obtained without stop codons for the contig sequences using NCBI ORF finder.

Phylogenetic analysis using neighbor joining (NJ) trees were constructed using sequences retrieved from NCBI GenBank (accession numbers are shown in the branch tips along with the sequence name) after ClustalW multiple alignment [14]. The tree was constructed using Kimura 2-parameter model in Mega (Molecular Evolutionary Genetics Analysis) version 10.0.1 [15]. The bootstrap value was adjusted as 1000. The outgroup we used for phylogenetic inferences was* Pseudomonas putida* (FJ404470.1)

### 2.9. Hydroponics

Strength seedlings were transferred to sterilized boiling tubes which were filled with 1/4 strength sterilized nitrogen-free Hoagland solution. Each plant was inoculated with 1ml of 2-week-old inocula. Four were replicated in total for each inoculum. Root length, shoot length, number of leaves, and presence and absence of the nodules, were recorded weekly throughout two and a half months.

## 3. Results and Discussion

*Pueraria phaseoloides* has been termed as a promiscuous plant for rhizobial strains [5]. However, there is very little knowledge on the non-rhizobial inhabitants of root nodules for* Pueraria phaseoloides*. We have isolated here a nonrhizobial partner, which we have named as Sub1. Using BLASTn analysis of a* nifH *fragment, sequenced in sense and in antisense, the closest level of identity we can arrive at is 99%, which is adequate to assign this bacterium, a specific species name. The genus that the BLASTn search identified at 99% identity was from the species* Pseudacidovorax intermedius, *of which little information is available. There were no rhizobia species in the BLASTn listing, demonstrating that this was a nonrhizobial contender, living inside the nodule of* Pueraria phaseoloides* plants. A bacterial strain, GAU_11_^T^, isolated from Japanese soils, has been shown to possess a sequence identity of 96.4% in 16s rRNA coding sequence to* Pseudacidovorax intermedius *CC_21_^T^ [19]. The* Pseudacidovorax *genus belongs to the family, Comamonadaceae, all members of the class beta-proteobacteria.

*NifH* sequences are divided into four clusters: cluster 1 containing Cyanobacteria,* Frankia*, Proteobacteria, and some clostridia, bacilli, and Nitrospirae; cluster 2 comprising vnfH nitrogenases; cluster 3 forming archaea and anaerobic bacteria; cluster 4 containing uncharacterized and nonfunctional* nifH* genes, which makes the* nifH *sequence of this study as belonging to cluster 1 [20]. The majority of the* nifH* sequences in public databases contain sequence data in the range between positions 100 and 500 bases (positions relative to* Azotobacter vinelandii*), which makes* nifH* sequences between this range, a good source to find suitable sequences, using nucleotide BLAST analysis [20].

In a comparative study that assessed sequence divergence levels of* nifH* against that of 16s rDNA, the authors found out that >97% similarity in 16S rRNA genes can be as much as 23% dissimilarity/divergence in* nifH* sequences, which tell us that* nifH* is less conserved than 16s rDNA [21]. Furthermore, in the same study, the authors show that ~80% of nitrogen-fixing strains that have >97% 16S rDNA identity and possess <95%* nifH* identity, and 43% are candidates for <85% identity, again verifying that* nifH* presents a window of opportunity to zoom into molecular identities, which cannot be resolved by 16s rDNA genes [21]. Our 99% sequence identity of* nifH,* to strains of* Pseudacidovorax*, tells us that a 99% identity of* nifH* is a stronger validation than the same match of 16s rDNA sequence; i.e., 99% identity of an otherwise variable* nifH* is proof that this sequence indeed belongs to the genus* Pseudacidovorax*.

Several nodulation-competent nonrhizobial species, belonging to *α* and *β* subgroups of Proteobacteria such as* Methylobacterium, Blastobacter, Devosia, Phyllobacterium, Ochrobactrum, Agrobacterium, Cupriavidus, Herbaspirillum, *and* Burkholderia* and some *δ*-Proteobacteria have been identified this far [2]. Although universally nitrogen fixation and nodulation genes have shown to be not strongly divergent, there are existing questions on the nodular nonrhizobial nitrogen-fixers due to sequence disparities in the nitrogen fixing (*nifH*) and nodulation (*nodD* or* nodA*) gene sequences, between Rhizobia and nonrhizobial nodule inhabitants [2].

**Figure 1:**
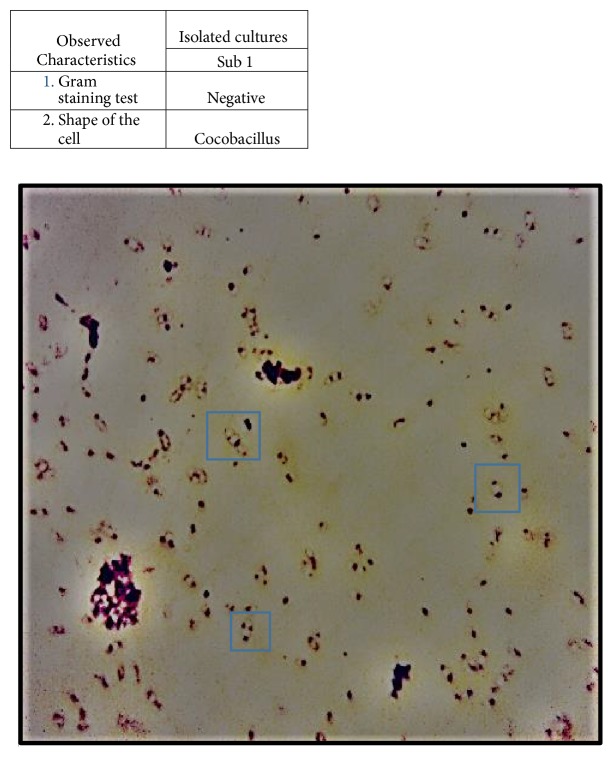
Light microscopy observations of Sub1 cultures. The coccobacillus cell shapes are pointed by blue boxes (above). Note: the image has been edited to sharpen the cell contours and is not a reflection upon Gram staining.

The shape of Sub1 cells was not rod shaped, as found in Rhizobia, but showed curved edges and was of a coccobacillus cell shape (Figure 1). This was further evidence that this was a nonrhizobial genus. The cells stained gram-negative and showed polarized bodies on opposite poles (Figure 1). The Sub1 cultures were able to degrade cellulose and milk protein, but not pectin (Table 2/Supplementary Figure 1).

The nodulation potential of Sub1 was tested in a hydroponics system, where 4 replicates were used to assess the ability of reinfection with Sub1, to induce nodule formation. There are many studies that have employed hydroponics to study legume nodulation [22–24] and, though different from soil, can be a better option in terms of root and nodule development; since there is no steric interference for abundant root growth, observations can be done easily and require less invasive means of harvesting than soil grown plants. There is even an innovative system called Rhizoponics (hydroponic rhizotron), which allows for the detailed study of root system architecture (RSA) [25].

**Figure 2:**
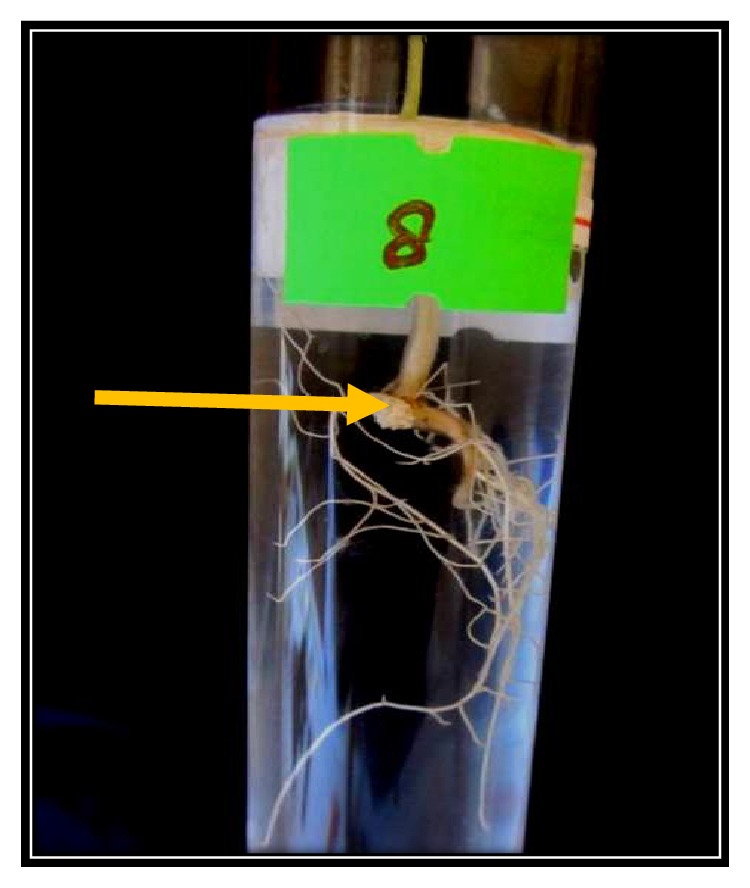
The formation of nodules by a replicate treated with Sub1 culture. The control plants are shown in Supplementary Figure 2.* The nodule (the enlarged area in peach color) found at the helm of the roots is shown with an orange arrow.*

There was early nodule formation of* Pueraria phaseoloides* plants treated with only Sub1. By week 4, all single plant replicates had shown development of nodules, indicating that this bacterium was capable of nodule formation in plants of* Pueraria phaseoloides* (Table 3/Figure 2). The reinfection by Sub1 too induced shoot elongation, compared to an equivalent negative control, at the end of the same time interval (Table 4). The nodules of the plant we took for isolation had large, spherical, and smooth nodules, both at the apex of the root and along the root, while the Sub1 infected nodules were peach white in color, were not spherical, and showed no pink color as seen in nodules infected by Rhizobia (Supplementary Figure 2). The nodules of the Sub1 infected plants were found at the helm and were not found as beads on the root. We witnessed the above characteristics based on observation of nodule features, as seen by the human eye while counting the nodules, week by week.

It has been shown that* nod* factors can induce leghemoglobin synthesis, before nitrogen fixation occurs and are thought to be a measure for the early stages of infection and not necessarily evidence of nitrogen fixation [26]. Still, there are deficiencies in nitrogenase activity in bacteria absent of leghemoglobin [26]. One of the measures of the presence of leghemoglobin is the color on the outside and the inside of the nodule. We, though, not observing pink color on the outside, can see red color on the interior of the cut nodule. Here, though, we cannot give a definite conclusion on the absence or presence of leghemoglobin in* Pueraria* plant nodules.

Sub1, which has the most number of nodules (3 per plant) from among four distinct isolates of* Pueraria phaseoloides* and has short roots, suggesting the “phytohormone and genetic contribution” that triggered prolific nodule formation in Sub1, interfered with root growth. The phytochemicals that play roles in the symbiotic trade-off between nodulation and root length are yet to be characterized thoroughly, although it is generally accepted that both local and systemic effects are crucial to the interrelationship between nodulation and root growth. The trafficking of photosynthate, the regulation performed by carbon stocks, and some level of genetic involvement, with genes such as* Cell Division Cycle 16* gene, too are crucial determinants of the relationship between nodule number and root length [27]. It has been further shown that nodulation (nodule number) and root length are inversely linked, which is also demonstrated in our study [28]. The controls in the absence of nodules have longer roots and not the Sub1 infected plants (Supplementary Figure 4), and when we use statistics (one-way ANOVA) from week to week, to compare the differences in root length between test and control, we observe that, at 6th week after reinfection, there is a statistically significant different (P<0.05) in root length between Sub1 treated plants and the negative controls (Supplementary Table 1).

Sub1 infected hydroponic plants show elongation of shoots, significantly stronger than the control plants (Supplementary Figure 4) and the shoot length difference was statistically significant at week 7 (Supplementary Table 2), which demonstrated that there was a spike in shoot development induced by Sub1 that was deficient in the control plants. We suggest cautiously, that this type of growth can be due to phytohormone secretion, or perhaps even genetic contributions. Furthermore, Sub1 infected plants possessed less emergent leaves compared to the negative controls (Table 4, Supplementary Figure 4)

Pseudonodules are outgrowths in roots, which are not instigated by Rhizobia or other bacterial contenders [29]. Pseudonodules have been shown to be induced by exogenous applied cytokinins, constitutive active cytokinin receptors, and synthetic auxin transport inhibition [29]. The absence of nodules in our negative control (Supplementary Figure 2) with no bacterial reinfection is evidence that the bacteria-mediated nodulation consists of real nodules and not pseudonodules; i.e., bacteria were the triggers and not any physiological factor.

**Figure 3:**
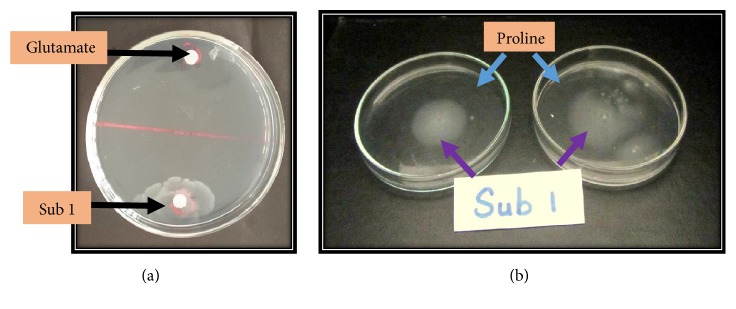
Chemotactic swarm plate for the assessment of movement of Sub1 towards glutamate through swarm behavior (a) and the swarming pattern of Sub1 to proline (b).

We also looked at the contenders for chemotaxis induction, using swarming assays. We tested proline, an amino acid inferred to be a chemotaxis inducer for* Rhizobium meliloti* [17]. Naringenin which in Rhizobia induced positive chemotaxis [30] and Quercetin, which has been identified as an inhibitory flavonoid and glutamate, are one of the most common amino acids found in plants. There was no swarming behavior shown for any out of naringenin, quercetin, and glutamate, although a characteristic swarming pattern (bulls eye) was seen, for when proline was in the medium (Figure 3). Swarming motility, as demonstrated here, has been shown to be restricted to three bacterial families, namely, Firmicutes, alpha, and gamma proteobacteria [31]. Sub1 belongs to beta proteobacteria and thus presents a novel family of bacteria shown to be conducive to swarming motility. The fact that Sub1 does not show swarming behavior to naringenin, quercetin, and glutamate does not mean that no other flavonoid or amino acid is incapable of inducing swarming motility.

The nodular inhabitants of* Pueraria mirifica* were all rhizobial, with 9 strains of the genus* Rhizobium* and 3 of* Bradyrhizobium* [32]. The exact nature of the nodular partners in* Pueraria phaseoloides *has not been studied this far and this is why we explored the microbiome of nodules, of this widely used cover crop. With Sub1, we have also isolated 3 other nodular bacteria, which we have characterized using colony and cell morphology (data not shown).

**Figure 4:**
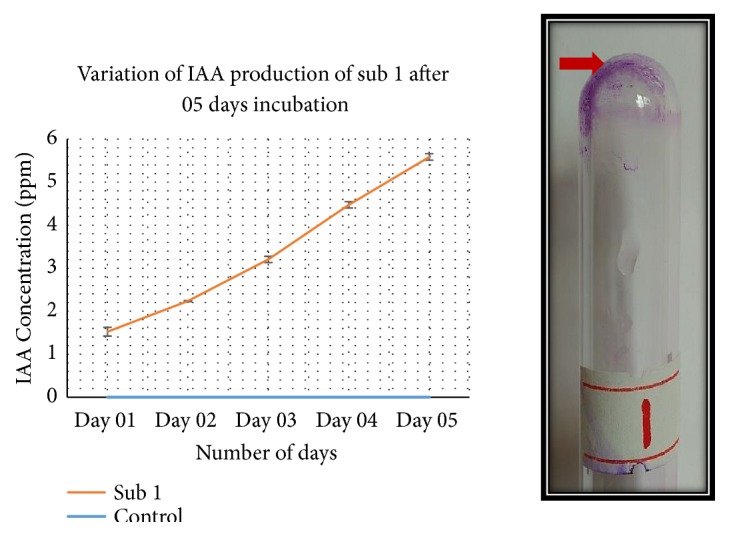
The development of purple color in tubes treated with crystal violet, indicative of biofilm formation (right), and the graph demonstrating the increase in IAA production in a period of 5 days by Sub1 cultures (left). The negative control of biofilm formation is provided in Supplementary Figure 5.* Note*: the control goes along the X-axis.

The ability of Sub1 to produce IAA to the extracellular medium makes the bacterium a good candidate for plant growth promotion, of which the observable feature was the higher induced shoot length compared to the negative uninfected control. Other benefits from IAA include, seed germination, development of xylem and root systems, mediation of responses to abiotic factors, photosynthesis, pigment formation, and resistance to stressful conditions [33]. The impact of IAA is plant-specific, since there are no advantages from IAA, to the bacterial cells. In a study performed by Kafrafi et al., 2017, they showed that in 7-day liquid bacterial cultures, the IAA levels were between 5 and 7 ppm [34]. This was analogous to our findings where we observed 5-6 ppm IAA in 5-day cultures (Figure 4). However, Sub1 was not able to solubilize inorganic calcium phosphate, demonstrating that it may not be secreting phosphate solubilizing inorganic acids, to the extracellular medium (data not shown).

It is known that the rhizosphere of a plant is a conducive environment for biofilm formation, with both moisture and nutrition for such changes. Rhizobia form biofilms in their capacities to infect the plant through infection threads and to form nodules [35]. The roles attributed to Rhizobia in biofilm formation include quorum sensing, infection, nutrition, and forming fungal associations [35]. Sub1 too has the potential to form biofilms as shown by the biofilm assays and may rely on biofilm formation in any of the processes where attachment to a surface may be a requirement (Figure 4).

**Figure 5:**
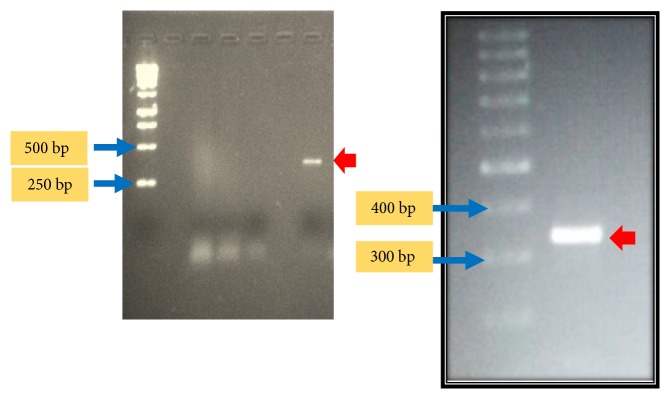
PCR amplification of* nifH* (left) and* nifD* (right) fragments using genomic DNA from Sub1 as template. The PCR amplicons of the correct sizes are indicated by red arrows.

We tested for the genomic DNA of Sub1 to possess the main* nif* genes,* nifH,* and* nifD*, in their genomes. The primers relevant to the two genes, both gave amplicons of the correct size, demonstrating that the primary* nif* gene architecture for nitrogen fixation, i.e.,* nifD* and* nifH*, was present in the genome of Sub1 (Figure 5). The bands were thick and solitary, showing that this could not have been nonspecific bands appearing in the PCR reaction. The* nifD* primers we employed were cyanobacterial biased, and yet they were able to amplify the* nifD* fragment, perhaps due to degenerate binding, to the* nifD* template. Nitrogen fixation is thought to be an ancestral function (before the evolution of legumes) that has been inferred to be receptive to horizontal gene transfer as well being lost from selective bacterial lineages [2]. Distinctly, It is also suggested that horizontal gene transfer of nodulation genes took place following the evolution of legumes, but the provider/s of the nodulation genes is/are yet unknown [2].

**Figure 6:**
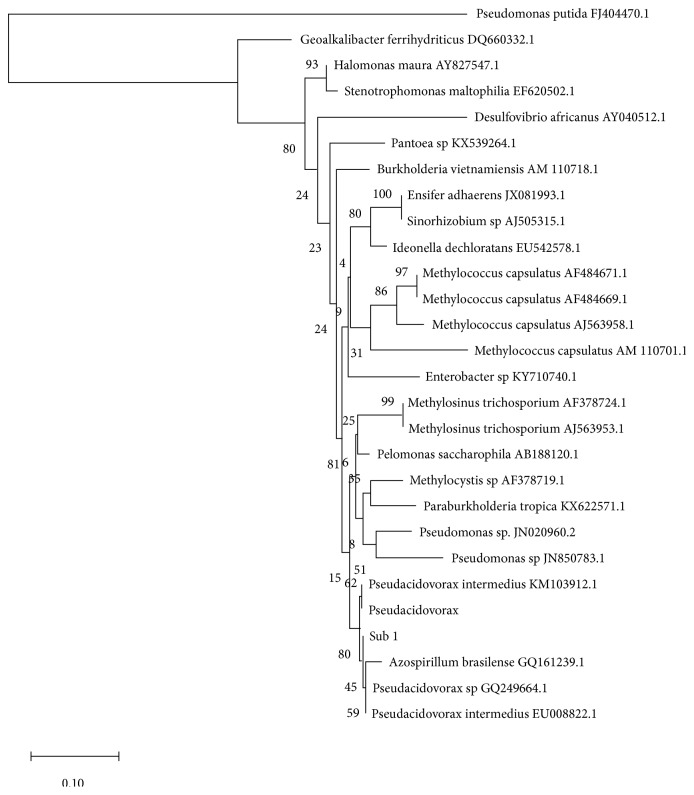
Phylogenetic analysis using neighbor Joining (NJ) trees was constructed using sequences retrieved from NCBI GenBank (accession numbers are shown in the branch tips along with the sequence name) after ClustalW multiple alignment [14]. The tree was constructed using Kimura 2-parameter model in Mega (Molecular Evolutionary Genetics Analysis) version 10.0.1 [15]. The bootstrap value was adjusted as 1000.

When we constructed a phylogenetic tree (*Neighborhood Joining*, Figure 6), with the nearest sequences of the BLASTn output, spanning four groupings of proteobacteria, alpha, beta, gamma, and delta, we found that four* Pseudacidovorax nifH* sequences clustering together with Sub1, in the phylogenetic tree, which too included a single* Azospirillum*. The fact that only one* Azospirillum *clusters with four* Pseudacidovorax*, tells us that the* Azospirillum* bacterium is either a unique anomaly or is a misclassification. Azospirilla bacteria are found as associative symbiotic rhizosphere inhabitants, belonging to the division, alpha proteobacteria and are known for their plant growth promotion through the production of phytohormones. As far as we know, there are no Azospirilla belonging to beta proteobacteria, while their presence is limited to the rhizosphere and the soil and are not known to form nodules or live inside them. We state that Sub1 belongs to the genus* Pseudacidovorax *and is likely a species of* Pseudacidovorax intermedius, *from sequencing of the* nifH *fragment, which was further confirmed through phylogenetic inferences.

## 4. Conclusions

- We have isolated here a nodulation-proficient bacterium, from the cover crop* Pueraria phaseoloides, *which is of a rare nonrhizobial identity, for a nodular inhabitant.
- The most significant finding of our study is the nodulation inducing potential of this nonrhizobial bacterium and its impact on shoot length.
- We state that this bacterium belongs to the species* Pseudacidovorax intermedius*, from the sequencing of a fragment of the* nifH* gene and its alignment, at 99% sequence identity, to* Pseudacidovorax intermedius* NH-1* nifH.*
- The phylogenetic tree constructed with the nearest neighbors of Sub1 provided a monophyletic cluster for the genus* Pseudacidovorax*, with one exception, an* Azospirillum* species.
- The bacterium was capable of secreting IAA, cellulases, and proteinases to the extracellular medium and contained the genetic foundation to produce the nitrogenase enzyme.
- Sub1 also showed some degree of biofilm formation, which could be helpful in the colonization of the nodules.

## Figures and Tables

**Table 1:** Primers used for the amplification of *nifD* and *nifH* genes in this study.

Gene	Primer sequence (5'-3')	Product size	Reference
*nifD*	nifD_F: CGGTTACTGGTCTTGGTCTGGTC nifD_R: GCGTCGTTAGCGATGTGGTGTC	338 bp	Glass et al. (2010) [6]
*nifH*	PolFor: TGCGACCCGAAGGCTGAC PolR: ATGGCCATCATCTCACCGGA	360 bp	Poly et al. (2001) [7]

**Table 2:** Cellulose, Pectin and Protein utilization by the isolated bacteria. Also see supplementary Figure 1 for pictures of plates.

Biochemical	Cellulose	Pectin	Protein
Sub 1	+	-	+

**Table 3:** The week by week changes in nodule number, in Sub1 treated hydroponic plants.

Treatment	18/09/15	1st week	2nd week	3rd week	4th week	5th week	6th week	7th week	8th week	9th week
1	0	0	0	0	1	1	1	1	1	1
1	0	0	0	1	2	2	2	3	3	4
1	0	0	0	0	3	3	5	5	5	5
1	0	0	0	0	2	2	3	3	3	3

**Table 4:** The hydroponics experiments to assess the contribution of Sub1 to plant growth parameters.

Treatment	Shoot length(cm) Mean ± SE	Root length(cm) Mean ± SE	Number of leaflets Mean ± SE	Number of nodules Mean ± SE
C	8.967 ± 0.567	15.000 ± 0.875	9.250 ± 0.289	0.000 ± 0.000
Sub 1	10.825 ± 0.788	8.68 ± 0.834	6.25 ± 3.25	3.000 ± 0.816

## Data Availability

The data used to support the findings of this study are available from the corresponding author upon request.

## Supplementary Materials

Supplementary Materials Supplementary Figure 1: cellulose, pectin, and protein utilization by Sub1. Supplementary Figure 2(a): original nodules that were used for the isolation process, as observed in Pueraria plants. Supplementary Figure 2(b): two negative controls of Sub1 with no visible nodules (above). Supplementary Figure 3: negative controls for proline plates to assess swarming motility by Sub1. Supplementary Figure 4: Sub1 infected plants (top) and control plants (bottom), showing differences in shoot length at week 5 after reinfection. Supplementary Figure 5: the negative control showing no biofilm formation. Biofilm formation by Sub1 is shown in Figure 4. Supplementary Table 1: statistical analysis on root lengths of the plants. Supplementary Table 2: statistical analysis of shoot lengths in plants.
