# Supplementary material for: A Nodulation-Proficient Nonrhizobial Inhabitant of Pueraria phaseoloides
Source: ScientificWorldJournal. 2019 Apr 1;2019:9782684. doi: 10.1155/2019/9782684 (PMC6463565; doi:10.1155/2019/9782684)
Supplement: Supplementary Materials — Supplementary Figure 1: cellulose, pectin, and protein utilization by Sub1. Supplementary Figure 2(a): original nodules that were used for the isolation process, as observed in Pueraria plants. Supplementary Figure 2(b): two negative controls of Sub1 with no visible nodules (above). Supplementary Figure 3: negative controls for proline plates to assess swarming motility by Sub1. Supplementary Figure 4: Sub1 infected plants (top) and control plants (bottom), showing differences in shoot length at week 5 after reinfection. Supplementary Figure 5: the negative control showing no biofilm formation. Biofilm formation by Sub1 is shown in Figure 4. Supplementary Table 1: statistical analysis on root lengths of the plants. Supplementary Table 2: statistical analysis of shoot lengths in plants. [file 9782684.f1.pdf]

Supplementary Figures and Tables

Cellulose utilization by Sub1.

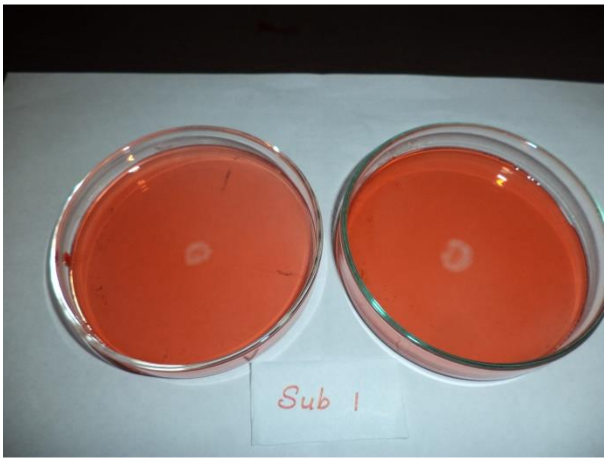

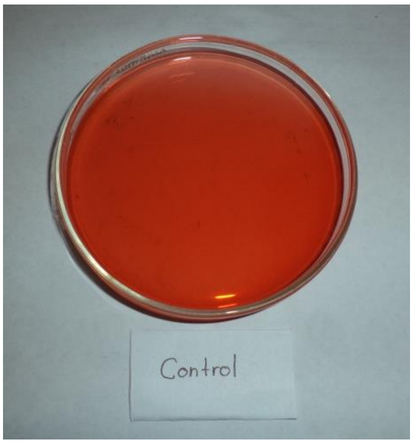

Protein utilization by Sub 1.

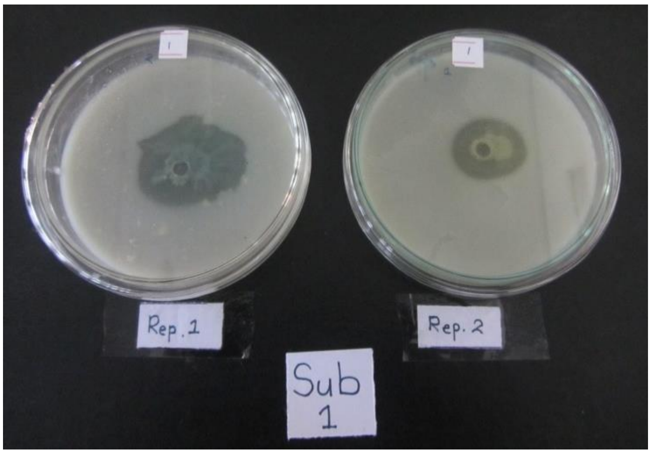

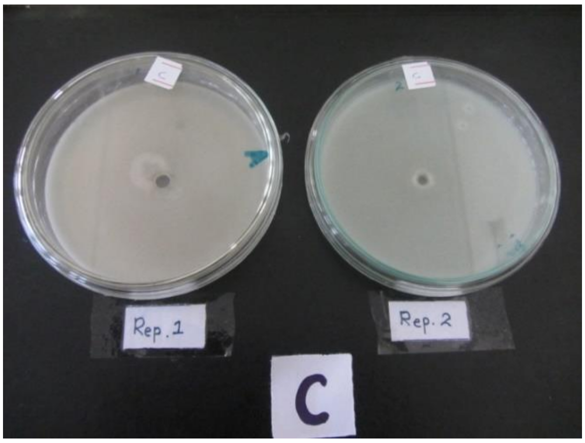

Pectin utilization by Sub1 (Test plates with no pectinase activity)

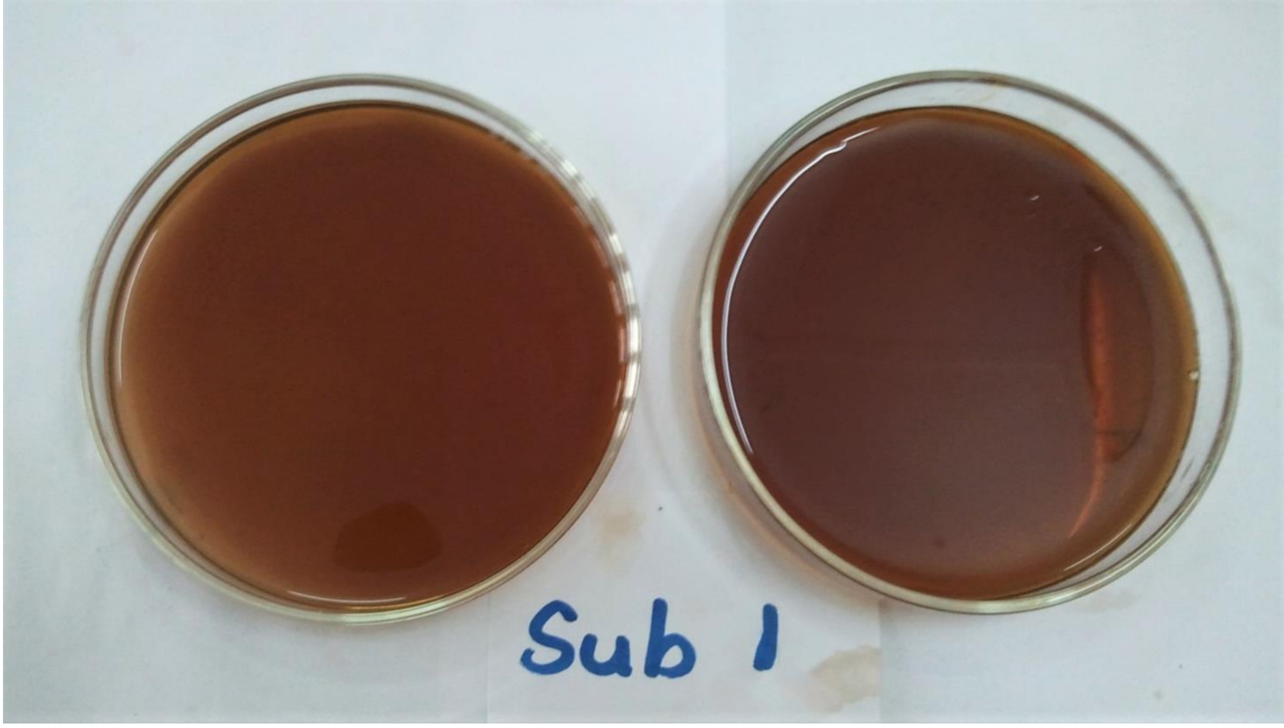

Supplementary Figure 1 – Cellulose, Pectin and Protein utilization by Sub1.

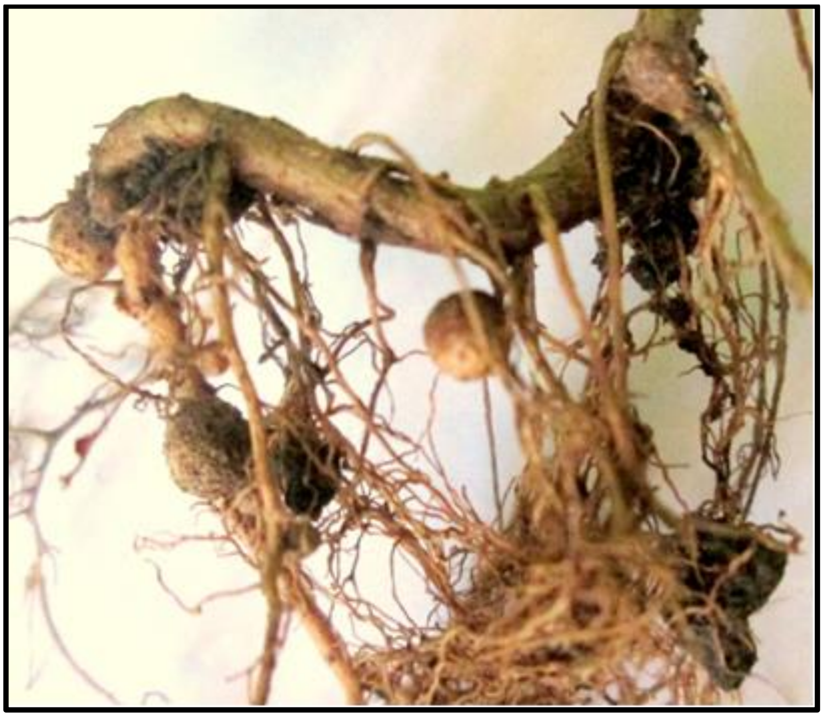

Supplementary Figure 2(a) - Original nodules that were used for the isolation process, as observed in *Pueraria* plants.

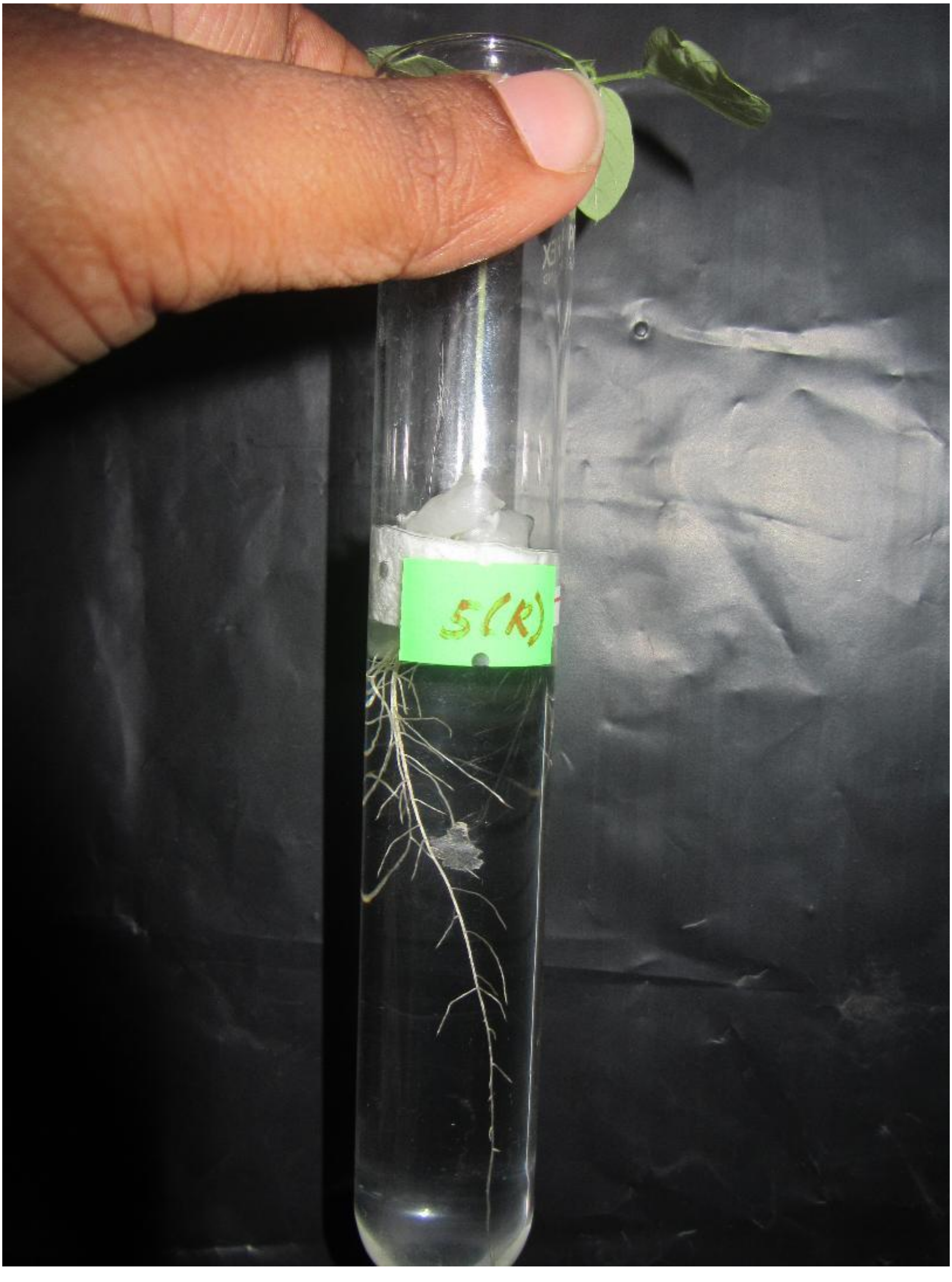
5(K)

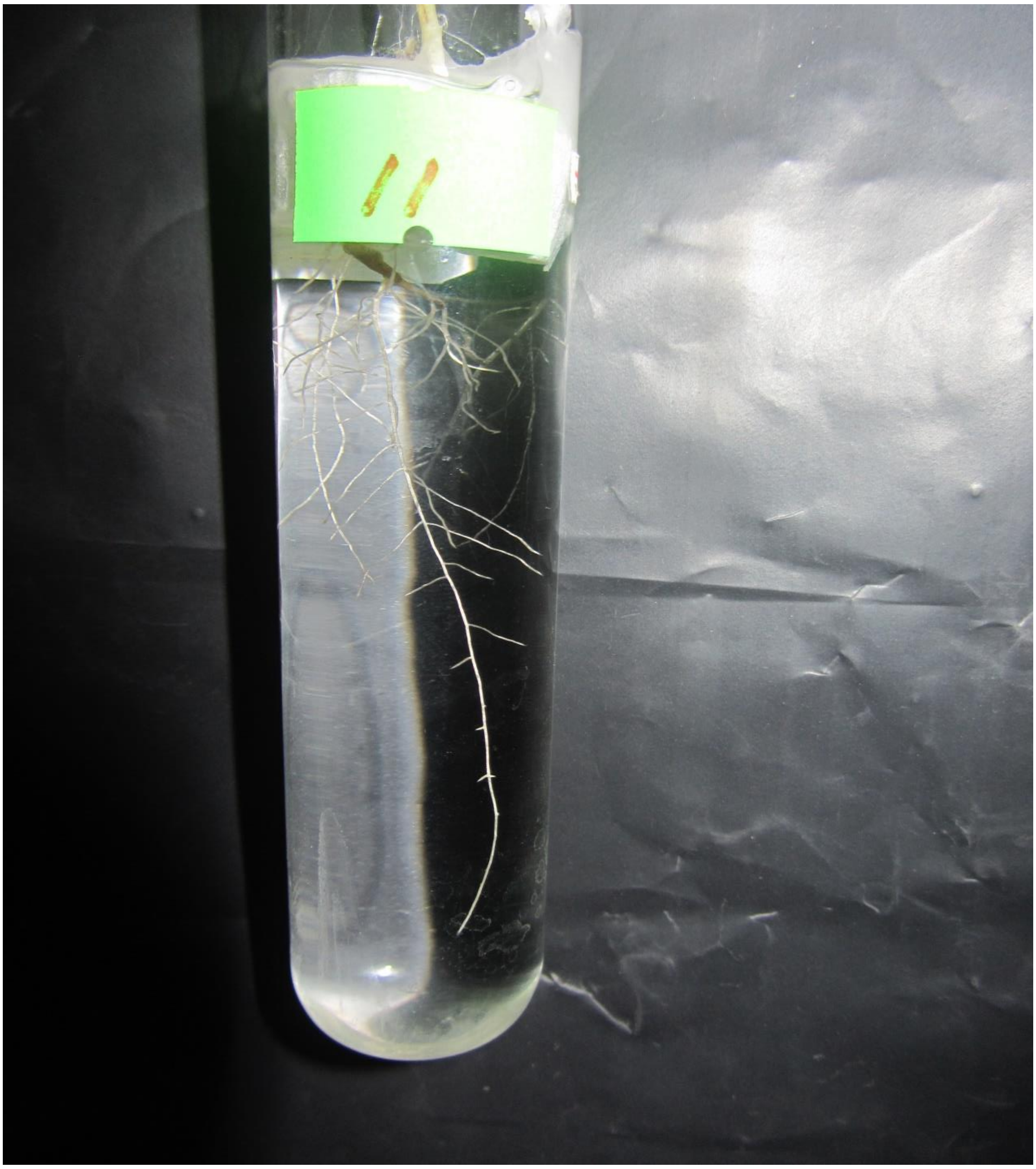

Supplementary Figure 2(b)- Two negative controls of Sub1 with no visible nodules (above)

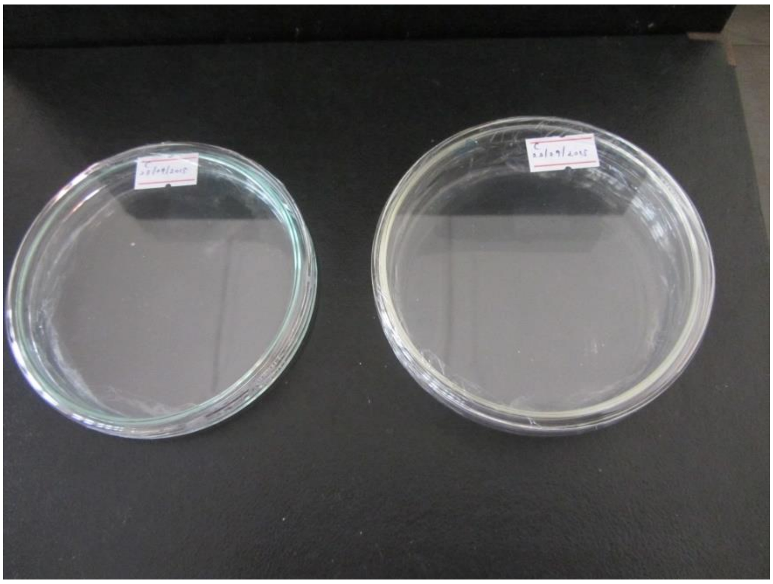

Supplementary figure 3 - Negative controls for proline plates to assess swarming motility by Sub1.

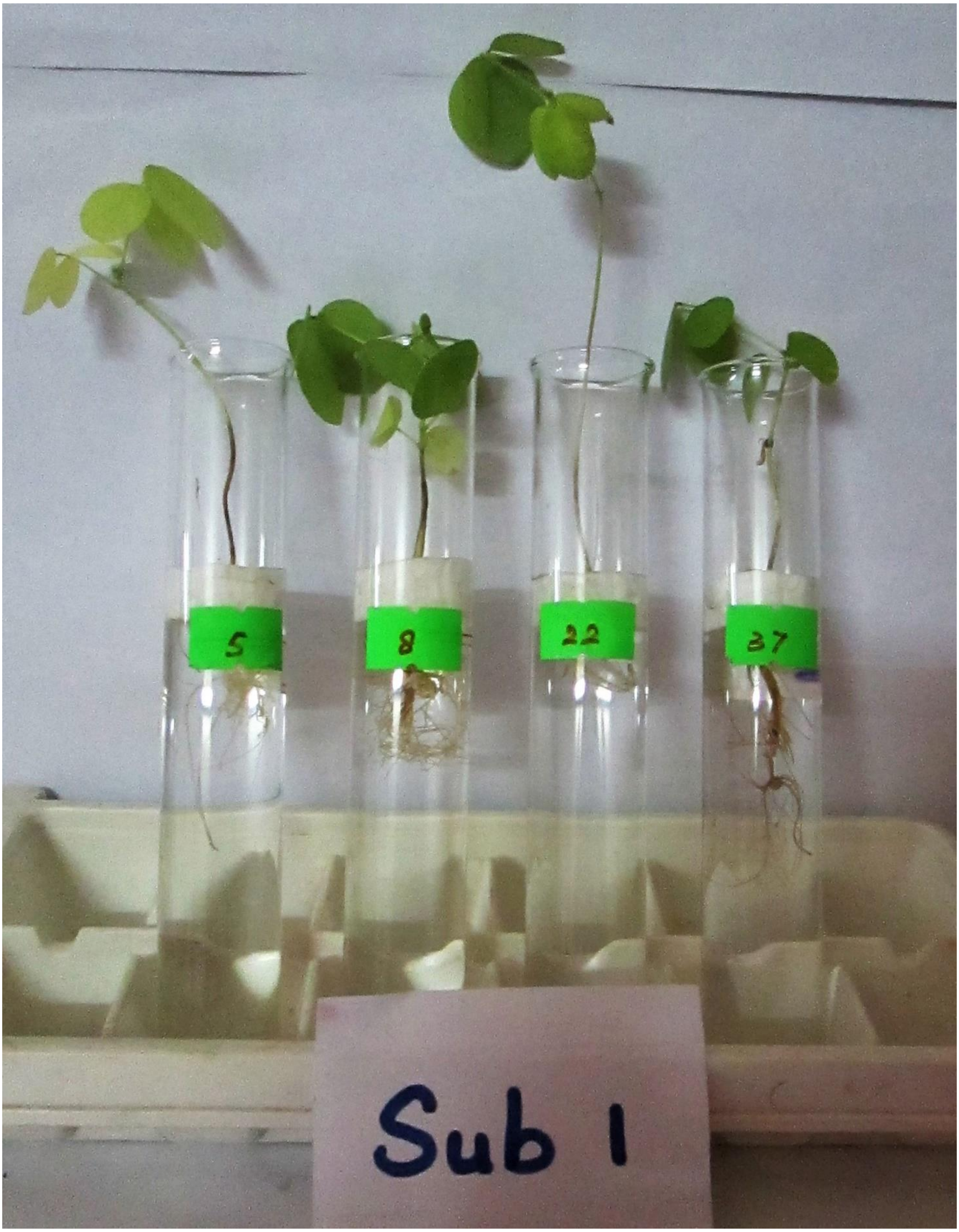
5
8
22
37
Sub 1

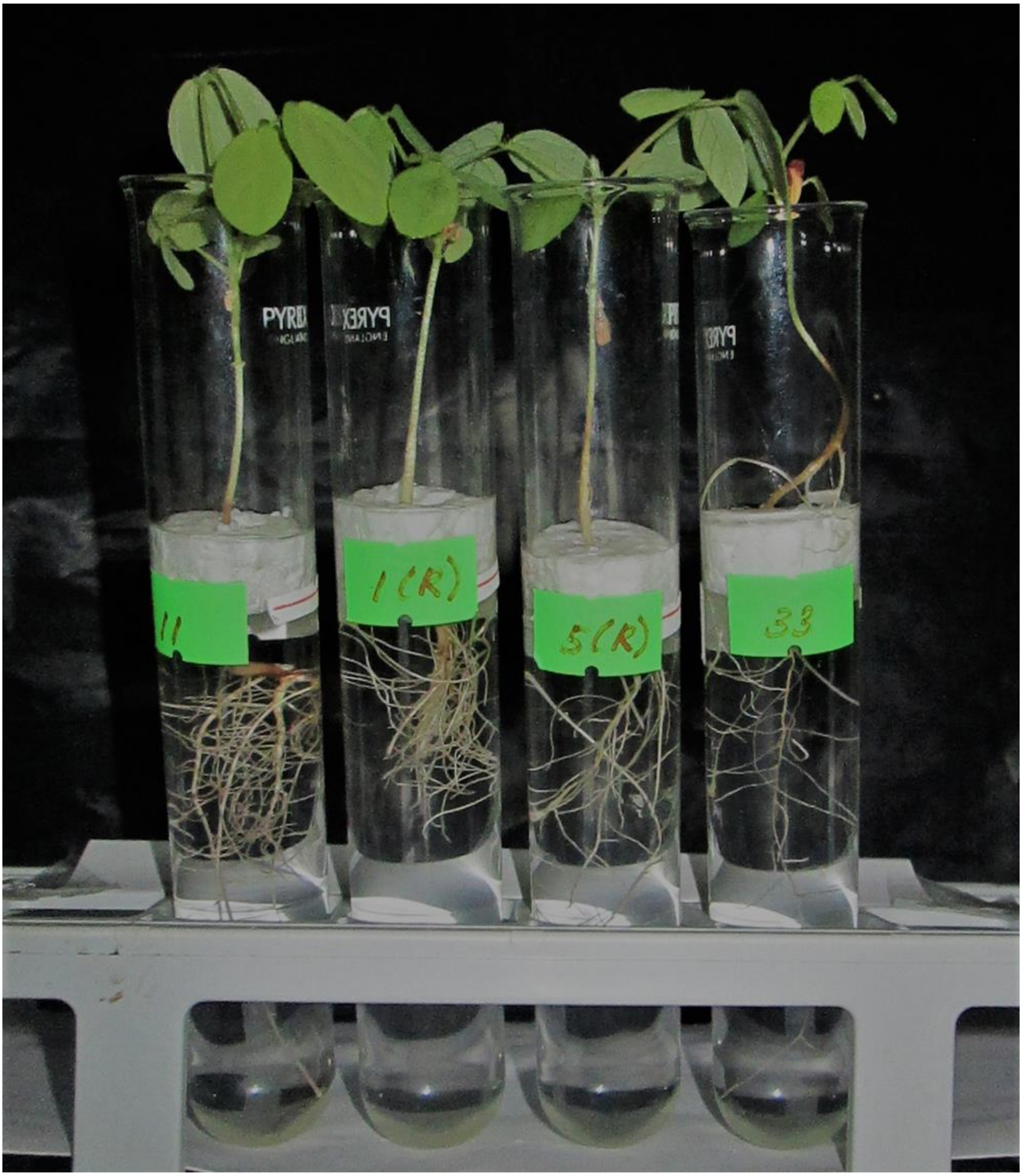

Supplementary Figure 4 – Sub1 infected plants (top) and control plants (bottom), showing differences in shoot length at week 5, post-reinfection.

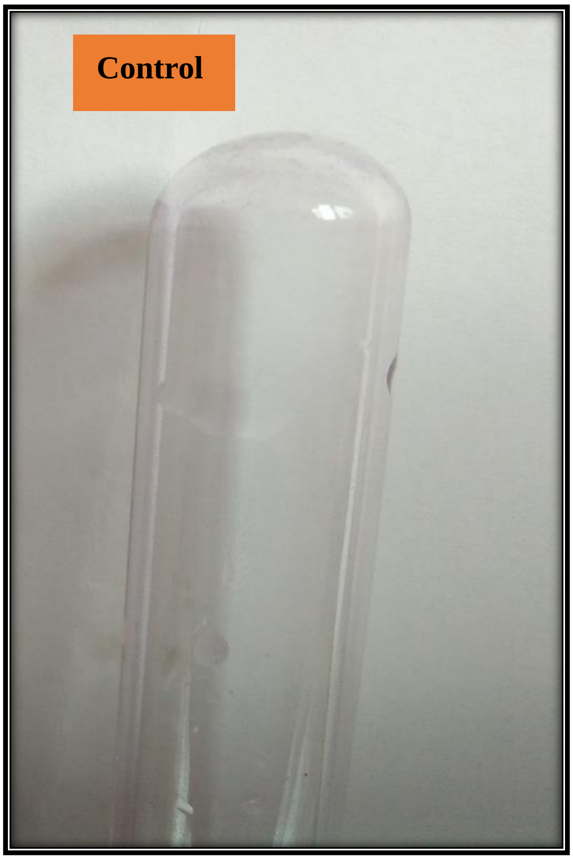

Supplementary Figure 5 – The negative control showing no biofilm formation. Biofilm formation by Sub1 is shown in figure 3.

Supplementary Table 1 – Statistical analysis on root lengths of the plants

One-way ANOVA: Begining_1 versus Experiment_1

```
Method

Null hypothesis         All means are equal
Alternative hypothesis  At least one mean is different
Significance level      α = 0.05

Equal variances were assumed for the analysis.


Factor Information

Factor        Levels  Values
Experiment_1       2  Control, Sub 1


Analysis of Variance

Source        DF  Adj SS  Adj MS  F-Value  P-Value
Experiment_1   1  0.6050  0.6050     2.30    0.180
Error          6  1.5750  0.2625
Total          7  2.1800


Model Summary

       S    R-sq  R-sq(adj)  R-sq(pred)
0.512348  27.75%     15.71%       0.00%


Means

Experiment_1  N   Mean  StDev       95% CI
Control       4  1.175  0.450  (0.548, 1.802)
Sub 1         4  1.725  0.568  (1.098, 2.352)

Pooled StDev = 0.512348
```

One-way ANOVA: 1st week_1 versus Experiment_1

```
Method

Null hypothesis         All means are equal
Alternative hypothesis  At least one mean is different
Significance level      α = 0.05

Equal variances were assumed for the analysis.


Factor Information

Factor        Levels  Values
Experiment_1       2  Control, Sub 1


Analysis of Variance

Source        DF  Adj SS  Adj MS  F-Value  P-Value
```

Experiment_1	1	0.1513	0.1513	0.16	0.707
Error	6	5.8175	0.9696		
Total	7	5.9687			

Model Summary

S	R-sq	R-sq(adj)	R-sq(pred)
0.984674	2.53%	0.00%	0.00%

Means

Experiment_1	N	Mean	StDev	95% CI
Control	4	2.275	1.328	(1.070, 3.480)
Sub 1	4	2.550	0.420	(1.345, 3.755)

Pooled StDev = 0.984674

One-way ANOVA: 2nd week_1 versus Experiment_1

Method

Null hypothesis	All means are equal
Alternative hypothesis	At least one mean is different
Significance level	α = 0.05

Equal variances were assumed for the analysis.

Factor Information

Factor	Levels	Values
Experiment_1	2	Control, Sub 1

Analysis of Variance

Source	DF	Adj SS	Adj MS	F-Value	P-Value
Experiment_1	1	0.02000	0.02000	0.01	0.912
Error	6	9.12000	1.52000		
Total	7	9.14000			

Model Summary

S	R-sq	R-sq(adj)	R-sq(pred)
1.23288	0.22%	0.00%	0.00%

Means

Experiment_1	N	Mean	StDev	95% CI
Control	4	3.500	1.294	(1.992, 5.008)
Sub 1	4	3.600	1.169	(2.092, 5.108)

Pooled StDev = 1.23288

One-way ANOVA: 3rd week_1 versus Experiment_1

Method

Null hypothesis	All means are equal
Alternative hypothesis	At least one mean is different
Significance level	α = 0.05

Equal variances were assumed for the analysis.

Factor Information

Factor	Levels	Values
Experiment_1	2	Control, Sub 1

Analysis of Variance

Source	DF	Adj SS	Adj MS	F-Value	P-Value
Experiment_1	1	0.1250	0.1250	0.05	0.830
Error	6	14.9150	2.4858		
Total	7	15.0400			

Model Summary

S	R-sq	R-sq(adj)	R-sq(pred)
1.57665	0.83%	0.00%	0.00%

Means

Experiment_1	N	Mean	StDev	95% CI
Control	4	5.225	1.115	(3.296, 7.154)
Sub 1	4	4.975	1.931	(3.046, 6.904)

Pooled StDev = 1.57665

One-way ANOVA: 4th week_1 versus Experiment_1

Method

Null hypothesis	All means are equal
Alternative hypothesis	At least one mean is different
Significance level	α = 0.05

Equal variances were assumed for the analysis.

Factor Information

Factor	Levels	Values
Experiment_1	2	Control, Sub 1

Analysis of Variance

Source	DF	Adj SS	Adj MS	F-Value	P-Value
Experiment_1	1	5.281	5.281	0.55	0.487
Error	6	57.808	9.635		
Total	7	63.089			

Model Summary

S	R-sq	R-sq(adj)	R-sq(pred)
3.10396	8.37%	0.00%	0.00%

Means

Experiment_1	N	Mean	StDev	95% CI
Control	4	8.82	3.45	(5.03, 12.62)
Sub 1	4	7.20	2.71	(3.40, 11.00)

Pooled StDev = 3.10396

One-way ANOVA: 5th week_1 versus Experiment_1

Method

Null hypothesis	All means are equal
Alternative hypothesis	At least one mean is different
Significance level	α = 0.05

Equal variances were assumed for the analysis.

Factor Information

Factor	Levels	Values
Experiment_1	2	Control, Sub 1

Analysis of Variance

Source	DF	Adj SS	Adj MS	F-Value	P-Value
Experiment_1	1	25.56	25.561	3.00	0.134
Error	6	51.19	8.531		
Total	7	76.75			

Model Summary

S	R-sq	R-sq(adj)	R-sq(pred)
2.92083	33.31%	22.19%	0.00%

Means

Experiment_1	N	Mean	StDev	95% CI
Control	4	11.48	3.31	(7.90, 15.05)
Sub 1	4	7.90	2.46	(4.33, 11.47)

Pooled StDev = 2.92083

One-way ANOVA: 6th week_1 versus Experiment_1

Method

Null hypothesis	All means are equal
Alternative hypothesis	At least one mean is different
Significance level	α = 0.05

Equal variances were assumed for the analysis.

Factor Information

Factor	Levels	Values
Experiment_1	2	Control, Sub 1

Analysis of Variance

Source	DF	Adj SS	Adj MS	F-Value	P-Value
Experiment_1	1	53.05	53.045	9.78	0.020
Error	6	32.53	5.422		
Total	7	85.58			

Model Summary

S	R-sq	R-sq(adj)	R-sq(pred)
2.32845	61.99%	55.65%	32.42%

Means

Experiment_1	N	Mean	StDev	95% CI
Control	4	13.40	2.48	(10.55, 16.25)
Sub 1	4	8.25	2.17	(5.40, 11.10)

Pooled StDev = 2.32845

One-way ANOVA: 7th week_1 versus Experiment_1

Method

Null hypothesis	All means are equal
Alternative hypothesis	At least one mean is different
Significance level	$\alpha = 0.05$

Equal variances were assumed for the analysis.

Factor Information

Factor	Levels	Values
Experiment_1	2	Control, Sub 1

Analysis of Variance

Source	DF	Adj SS	Adj MS	F-Value	P-Value
Experiment_1	1	80.01	80.011	20.81	0.004
Error	6	23.07	3.845		
Total	7	103.08			

Model Summary

S	R-sq	R-sq(adj)	R-sq(pred)
1.96076	77.62%	73.89%	60.22%

Means

Experiment_1	N	Mean	StDev	95% CI
Control	4	15.000	1.667	(12.601, 17.399)
Sub 1	4	8.68	2.22	(6.28, 11.07)

Pooled StDev = 1.96076

Supplementary Table 2 - Statistical analysis of shoot lengths in plants

One-way ANOVA: 1st week versus Treatment

Method

Null hypothesis	All means are equal
Alternative hypothesis	At least one mean is different
Significance level	α = 0.05

Equal variances were assumed for the analysis.

Factor Information

Factor	Levels	Values
Treatment	7	1, 2, 3, 4, 5, 6, 7

Analysis of Variance

Source	DF	Adj SS	Adj MS	F-Value	P-Value
Treatment	6	15.95	2.658	1.02	0.439
Error	21	54.67	2.603		
Total	27	70.62			

Model Summary

S	R-sq	R-sq(adj)	R-sq(pred)
1.61352	22.58%	0.46%	0.00%

Means

Treatment	N	Mean	StDev	95% CI
1	4	8.000	1.992	(6.322, 9.678)
2	4	7.050	1.863	(5.372, 8.728)
3	4	5.850	0.661	(4.172, 7.528)
4	4	7.225	1.130	(5.547, 8.903)
5	4	7.38	2.50	(5.70, 9.05)
6	4	6.825	0.714	(5.147, 8.503)
7	4	5.750	1.520	(4.072, 7.428)

Pooled StDev = 1.61352

One-way ANOVA: 2nd week versus Treatment

Method

Null hypothesis	All means are equal
Alternative hypothesis	At least one mean is different
Significance level	α = 0.05

Equal variances were assumed for the analysis.

Factor Information

Factor	Levels	Values
Treatment	7	1, 2, 3, 4, 5, 6, 7

Analysis of Variance

Source	DF	Adj SS	Adj MS	F-Value	P-Value
Treatment	6	13.50	2.250	0.91	0.505
Error	21	51.77	2.465		
Total	27	65.27			

Model Summary

S	R-sq	R-sq(adj)	R-sq(pred)
1.57003	20.69%	0.00%	0.00%

Means

Treatment	N	Mean	StDev	95% CI
1	4	8.35	2.06	(6.72, 9.98)
2	4	7.07	2.17	(5.44, 8.71)
3	4	7.000	0.770	(5.367, 8.633)
4	4	7.850	1.079	(6.217, 9.483)
5	4	8.250	1.984	(6.617, 9.883)
6	4	7.125	0.618	(5.492, 8.758)
7	4	6.300	1.494	(4.667, 7.933)

Pooled StDev = 1.57003

One-way ANOVA: 3rd week versus Treatment

Method

Null hypothesis	All means are equal
Alternative hypothesis	At least one mean is different
Significance level	$\alpha = 0.05$

Equal variances were assumed for the analysis.

Factor Information

Factor	Levels	Values
Treatment	7	1, 2, 3, 4, 5, 6, 7

Analysis of Variance

Source	DF	Adj SS	Adj MS	F-Value	P-Value
Treatment	6	12.72	2.120	0.90	0.510
Error	21	49.23	2.344		
Total	27	61.95			

Model Summary

S	R-sq	R-sq(adj)	R-sq(pred)
1.53107	20.54%	0.00%	0.00%

Means

Treatment	N	Mean	StDev	95% CI
1	4	8.77	2.10	(7.18, 10.37)
2	4	8.05	2.25	(6.46, 9.64)
3	4	7.775	0.834	(6.183, 9.367)
4	4	7.925	1.075	(6.333, 9.517)
5	4	8.500	1.766	(6.908, 10.092)
6	4	7.375	0.486	(5.783, 8.967)

```
7          4  6.575  1.305  (4.983,  8.167)

Pooled StDev = 1.53107
```

One-way ANOVA: 4th week versus Treatment

```
Method

Null hypothesis         All means are equal
Alternative hypothesis  At least one mean is different
Significance level      α = 0.05

Equal variances were assumed for the analysis.


Factor Information

Factor     Levels  Values
Treatment       7  1, 2, 3, 4, 5, 6, 7


Analysis of Variance

Source     DF  Adj SS  Adj MS  F-Value  P-Value
Treatment   6   18.37   3.062     1.38    0.269
Error      21   46.62   2.220
Total      27   64.99


Model Summary

      S    R-sq  R-sq(adj)  R-sq(pred)
1.48993  28.27%      7.77%       0.00%


Means

Treatment  N   Mean  StDev       95% CI
1          4   9.18   2.12  ( 7.63,  10.72)
2          4   8.63   2.43  ( 7.08,  10.17)
3          4  8.175  0.834  (6.626,  9.724)
4          4  8.150  0.819  (6.601,  9.699)
5          4  9.275  1.526  (7.726, 10.824)
6          4  7.450  0.473  (5.901,  8.999)
7          4  6.875  1.103  (5.326,  8.424)

Pooled StDev = 1.48993
```

One-way ANOVA: 5th week versus Treatment

```
Method

Null hypothesis         All means are equal
Alternative hypothesis  At least one mean is different
Significance level      α = 0.05

Equal variances were assumed for the analysis.


Factor Information

Factor     Levels  Values
Treatment       7  1, 2, 3, 4, 5, 6, 7


Analysis of Variance

Source     DF  Adj SS  Adj MS  F-Value  P-Value
```

Treatment	6	18.78	3.131	1.35	0.280
Error	21	48.74	2.321		
Total	27	67.53			

Model Summary

S	R-sq	R-sq(adj)	R-sq(pred)
1.52351	27.82%	7.19%	0.00%

Means

Treatment	N	Mean	StDev	95% CI
1	4	10.00	2.21	(8.42, 11.58)
2	4	9.22	2.42	(7.64, 10.81)
3	4	8.500	0.876	(6.916, 10.084)
4	4	9.450	1.318	(7.866, 11.034)
5	4	9.375	1.526	(7.791, 10.959)
6	4	7.725	0.479	(6.141, 9.309)
7	4	7.750	0.645	(6.166, 9.334)

Pooled StDev = 1.52351

One-way ANOVA: 6th week versus Treatment

Method

Null hypothesis	All means are equal
Alternative hypothesis	At least one mean is different
Significance level	$\alpha = 0.05$
Rows unused	1

Equal variances were assumed for the analysis.

Factor Information

Factor	Levels	Values
Treatment	7	1, 2, 3, 4, 5, 6, 7

Analysis of Variance

Source	DF	Adj SS	Adj MS	F-Value	P-Value
Treatment	6	22.28	3.714	1.72	0.168
Error	20	43.14	2.157		
Total	26	65.42			

Model Summary

S	R-sq	R-sq(adj)	R-sq(pred)
1.46866	34.06%	14.28%	0.00%

Means

Treatment	N	Mean	StDev	95% CI
1	4	10.325	1.960	(8.793, 11.857)
2	3	9.73	2.72	(7.96, 11.50)
3	4	8.700	0.898	(7.168, 10.232)
4	4	9.650	1.237	(8.118, 11.182)
5	4	9.500	1.472	(7.968, 11.032)
6	4	7.725	0.479	(6.193, 9.257)
7	4	7.975	0.943	(6.443, 9.507)

Pooled StDev = 1.46866

One-way ANOVA: 7th week versus Treatment

Method

Null hypothesis	All means are equal
Alternative hypothesis	At least one mean is different
Significance level	α = 0.05
Rows unused	2

Equal variances were assumed for the analysis.

Factor Information

Factor	Levels	Values
Treatment	7	1, 2, 3, 4, 5, 6, 7

Analysis of Variance

Source	DF	Adj SS	Adj MS	F-Value	P-Value
Treatment	6	26.21	4.369	2.72	**0.044**
Error	19	30.47	1.604		
Total	25	56.69			

Model Summary

S	R-sq	R-sq(adj)	R-sq(pred)
1.26640	46.24%	29.27%	0.00%

Means

Treatment	N	Mean	StDev	95% CI
1	4	10.825	1.576	(9.500, 12.150)
2	3	10.30	1.93	(8.77, 11.83)
3	4	8.975	0.967	(7.650, 10.300)
4	4	9.800	1.449	(8.475, 11.125)
5	3	8.967	0.981	(7.436, 10.497)
6	4	7.750	0.507	(6.425, 9.075)
7	4	8.500	1.122	(7.175, 9.825)

Pooled StDev = 1.26640

7th week of shoot length data sets were shown significant difference comparatively other weeks.
